# Home-based telerehabilitation after CABG: higher objectively verified adherence and 12-week functional outcomes in a single-center observational cohort

**DOI:** 10.3389/fcvm.2026.1947232

**Published:** 2026-10-15

**Authors:** Yan Liu, Xugang Wang, Cuihua Wang, Minjuan Zhang, Manman Kong, Meng Wang, Jingjing Ma, Huili Liu

**Affiliations:** 1 Department of Cardiovascular Surgery, The First Hospital of Hebei Medical University, Shijiazhuang, China; 2 Department of Cardiac Function Rehabilitation, The First Hospital of Hebei Medical University, Shijiazhuang, China; 3 Department of Otorhinolaryngology Head and Neck Surgery, Shijiazhuang People’s Hospital, Shijiazhuang, China; 4 Department of Thoracic Surgery, The First Hospital of Hebei Medical University, Shijiazhuang, China

**Keywords:** adherence, cardiac rehabilitation, coronary artery bypass grafting, observational study, routinely collected health data, telerehabilitation

## Abstract

**Background:**

Home-based telerehabilitation (HBTCR) may address access barriers to center-based cardiac rehabilitation (CBCR) after coronary artery bypass grafting (CABG). However, real-world comparative evidence on objectively verified adherence and functional recovery, with careful confounding control, remains sparse. This observational cohort study compared session completion and 12-week functional outcomes between HBTCR and CBCR in post-CABG patients using routine clinical data.

**Methods:**

This single-center observational cohort screened 288 patients and identified 195 potentially eligible post-CABG patients (February 2024–February 2025). After excluding 28 patients with unresolved rehabilitation exposure, 167 patients (56 HBTCR, 54 CBCR, 57 usual care) formed the propensity-score estimation cohort, adjusted for 25 baseline covariates with generalized propensity scores and overlap weighting. The primary complete-case analysis included 105 patients (35 per group) with complete 12-week follow-up. The primary outcome was objectively verified session completion (% of 36 prescribed sessions). The key clinical outcome was 12-week VO_2_peak, analyzed via weighted ANCOVA. Results are reported as adjusted differences with 95% CIs.

**Results:**

After weighting, covariates were well balanced (all SMDs <0.10). HBTCR showed significantly higher objectively verified session completion than CBCR (87.7% vs. 78.5%; overlap-weighted difference 9.1 percentage points, 95% CI: 4.4 to 13.7; *P* < 0.001). The adjusted HBTCR–CBCR difference in 12-week VO_2_peak was small and not statistically significant (0.17 mL/kg/min, 95% CI: −0.50 to 0.84; *P* = 0.616). Both active rehabilitation groups showed substantially larger 12-week VO_2_peak gains than usual care (adjusted differences >4.0 mL/kg/min).

**Conclusion:**

Among post-CABG patients who chose HBTCR or CBCR in routine practice, those choosing HBTCR had higher objectively verified session completion and comparable observed 12-week improvement in VO_2_peak. These findings support HBTCR as a feasible option for patients who prefer or require home-based rehabilitation because of work, travel, or scheduling constraints. Formal equivalence was not tested, and the clinical meaningfulness and cost-effectiveness of this delivery model warrant further evaluation.

## Introduction

Coronary artery disease (CAD) remains a leading cause of global morbidity and mortality, with coronary artery bypass grafting (CABG) the standard revascularization strategy for patients with complex, multivessel, or left main disease (1, 2). While CABG successfully restores myocardial blood flow and alleviates symptoms, the long-term prognosis depends on comprehensive secondary prevention strategies that address underlying risk factors and promote physiological recovery (3). Cardiac rehabilitation (CR), a multidisciplinary intervention that includes structured exercise, education, and psychosocial support, is a Class IA recommendation in international guidelines for post-CABG patients (4, 5).

The traditional CR model is center-based (CBCR), providing supervised exercise sessions in a hospital or outpatient setting. The benefits of CBCR are well-documented, including improvements in exercise capacity, cardiac function, and psychosocial well-being (6–8). However, real-world participation remains low: fewer than 50% of eligible patients in developed countries and less than 10% in developing nations attend a CR program (9, 10). Key barriers include geographical distance, transportation challenges, scheduling conflicts, and costs (11, 12).

Home-based telerehabilitation (HBTCR) has been proposed as a solution to these accessibility barriers, using wearable sensors, mobile applications, and video conferencing to deliver remotely monitored CR programs (13–17). Several systematic reviews and meta-analyses of randomized controlled trials (RCTs) have suggested that HBTCR can achieve clinical outcomes comparable to those of traditional CBCR in various cardiovascular populations (7, 18). A 2023 Cochrane review systematically evaluated both delivery modes, and a 2022 real-world study of over 2,500 patients compared home-based and center-based CR in routine practice (18, 19). A 2024 RCT further examined remote rehabilitation specifically in CABG patients (14).

Despite this evidence, several important questions remain. Most comparisons have been conducted in controlled trial settings where adherence may not reflect routine clinical behavior. Adherence has often been measured using different recording mechanisms across delivery modes, making direct comparison problematic. Observational studies have rarely controlled for the full range of confounders that influence both modality selection and outcomes, such as digital literacy, family support, and geographical accessibility. The present study was designed to address these implementation questions using routinely collected health data from a single center, with explicit attention to ascertainment bias, confounding control, and missing data.

The primary implementation objective was to compare objectively verified session completion between HBTCR and CBCR over 12 weeks. The key clinical objective was to estimate the adjusted between-group difference in VO_2_peak at 12 weeks, with comparisons against usual care serving as supportive analyses. Secondary objectives included comparisons of 6-minute walk distance (6MWD), left ventricular ejection fraction (LVEF), psychological status, and quality of life. We hypothesized that HBTCR would be associated with higher session completion than CBCR. The VO_2_peak analysis was estimation-focused; no equivalence or non-inferiority margin was prespecified.

## Relationship to prior reports

This analysis draws on data from patients who received cardiac rehabilitation at the First Hospital of Hebei Medical University. A previously reported randomized trial from our center examined home-based versus center-based cardiac rehabilitation in 60 post-CABG patients (30 per group) recruited between 2019 and 2020, focusing on psychological and quality-of-life outcomes (20). The present cohort was assembled from consecutive patients undergoing CABG between February 2024 and February 2025; cross-checking of surgical dates and program enrollment records against the trial enrollment log confirmed that no patient participated in both studies (0 of 60 trial participants). The present analysis further differs in its focus on objectively verified adherence, expanded confounder adjustment, and implementation-oriented research questions. Participant overlap, previously reported variables, and analyses unique to the present report are explicitly detailed in Supplementary Table S1.

## Methods

### Study design and setting

This was a single-center, observational cohort study conducted at the First Hospital of Hebei Medical University, a tertiary academic medical center in Shijiazhuang, China, using routinely collected electronic health records (EHR). The study is reported in accordance with the STROBE statement (21), the RECORD statement (22), and the TIDieR checklist (23). The completed checklists are provided in the Supplementary Materials. The study protocol was approved by the Institutional Ethics Committee of The First Hospital of Hebei Medical University (Reference number: S00775). For this secondary analysis of de-identified EHR data, the requirement for individual informed consent was waived.

### Study population

We retrospectively screened the EHR of all adult patients (aged 30–75 years) who underwent isolated CABG between February 2024 and February 2025. Initial eligibility required: (1) isolated CABG without another major cardiac procedure; (2) absence of severe postoperative complications precluding rehabilitation participation; (3) baseline LVEF ≥30%; and (4) no end-stage renal disease. Among 195 potentially eligible patients, rehabilitation exposure could not be resolved for 28 because of unclear or mixed modality documentation; the remaining 167 patients with clearly documented exposure (56 HBTCR, 54 CBCR, 57 usual care) constituted the propensity-score estimation cohort. Availability of a 12-week reassessment was not required for inclusion in this exposure cohort; absence of a 12-week assessment was treated as outcome missingness. The primary complete-case outcome analysis included the 105 patients (35 per group) with complete 12-week assessments, while missing-outcome sensitivity analyses were conducted in the 167 patients with resolved exposure.

### Time zero, exposure assignment, and follow-up

The index date (time zero) was defined as the date of the baseline CPET or, for usual-care patients, the corresponding postoperative outpatient assessment, occurring 4–8 weeks after CABG. For HBTCR and CBCR, the baseline assessment preceded the first rehabilitation session. Rehabilitation modality was assigned according to the initially documented program enrollment and was not reclassified according to subsequent adherence, completion, or crossover. Follow-up outcomes were assessed 12 weeks (±2 weeks) after the index date (Figure 1).

**Figure 1:**
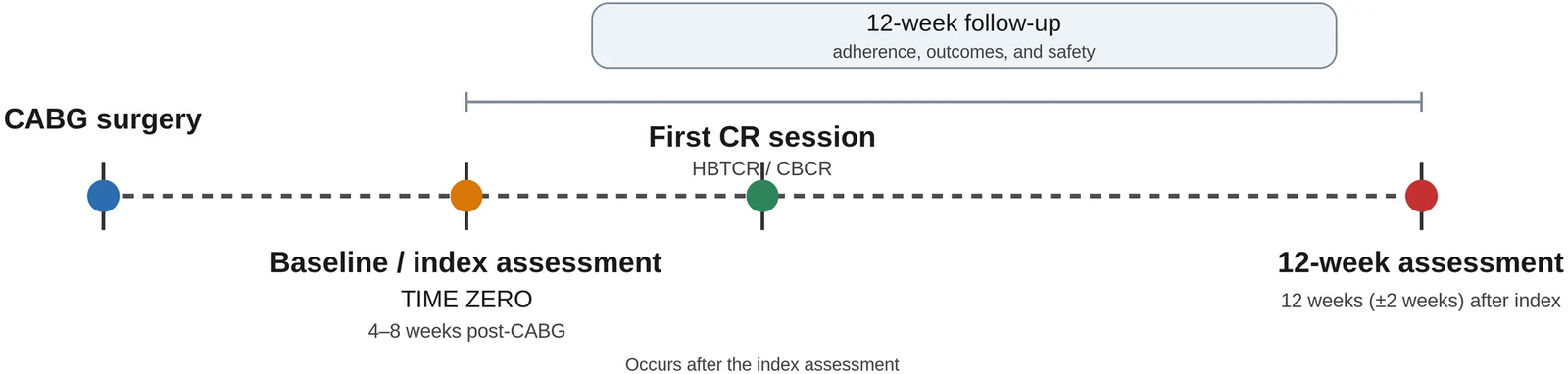
Study timeline and time-zero definition. The index date (time zero) was the baseline CPET or corresponding postoperative outpatient assessment performed 4–8 weeks after CABG. For HBTCR and CBCR, the first rehabilitation session occurred after the index assessment. Twelve-week outcomes were assessed 12 weeks (±2 weeks) after the index date.

### Rehabilitation modalities

Home-Based Telerehabilitation (HBTCR). Patients received a personalized exercise prescription and were provided with a wearable heart rate monitor (Polar H10) paired with a dedicated mobile application. The program consisted of three prescribed sessions per week for 12 weeks (36 sessions), each targeting 30–60 min of aerobic exercise at 60%–80% of heart rate reserve, plus resistance training (two sessions per week, 1–2 sets of 10–15 repetitions for major muscle groups at moderate intensity). Intensity was progressively increased every 2 weeks based on heart rate response and RPE (12–14 on the Borg scale). Home exercise sessions were performed independently without real-time audiovisual supervision; heart-rate data were recorded by the wearable device for objective verification. Scheduled video or telephone consultations were conducted approximately once weekly to review symptoms, adherence, and accumulated training data and to adjust the exercise prescription when clinically indicated. Abnormal heart rate alerts triggered immediate telephone contact.

Center-Based Cardiac Rehabilitation (CBCR). Patients attended the hospital's outpatient CR program with the same exercise prescription, progression criteria, and educational content as HBTCR, with the principal difference being supervised, in-person delivery with continuous ECG telemetry monitoring. Each of the 36 CBCR exercise sessions was directly supervised on site by rehabilitation staff, whereas HBTCR participants exercised independently at home and received approximately 12 scheduled tele-consultations. The two programs therefore differ not only in setting but also in the intensity of supervision and professional contact, and the present comparison evaluates the two delivery models as implemented in routine practice rather than equivalent programs administered in different settings.

#### Usual care (control)

Patients received standard post-operative follow-up without enrollment in any formal CR program.

### Data collection and covariates

Data were extracted from the EHR by two independent researchers. Covariates included demographics and anthropometrics; smoking and cardiovascular comorbidities; surgical and postoperative characteristics; baseline LVEF, VO_2_peak, and 6MWD; education, employment, urban residence, and distance to the rehabilitation center; smartphone/internet access and digital literacy; family support; travel barriers and scheduling constraints; NYHA class and Clinical Frailty Scale score; laboratory variables; and discharge medications. Variables not captured in the EHR were treated as potential unmeasured confounders rather than inferred.

### Outcome measures

Primary Implementation Outcome. Objectively verified session completion (adherence), defined as the percentage of completed rehabilitation sessions out of 36 prescribed, with a session considered completed only when at least 30 min of prescribed exercise were objectively documented at target intensity. For HBTCR, wearable device timestamps and heart rate recordings served as verification; app entries without valid wearable data were not counted. For CBCR, physiotherapist-signed attendance records and telemetry logs were used. Detailed implementation metrics are reported in Supplementary Table S2.

#### Key clinical outcome

The 12-week VO_2_peak (mL/kg/min), measured by symptom-limited CPET on a cycle ergometer with a ramp protocol of 10–15 W/min. VO_2_peak was defined as the highest 20-second averaged oxygen uptake. Lower VO_2_peak is strongly associated with increased mortality (24–26). Equipment was calibrated before each test, and outcome assessors were blinded to rehabilitation modality.

Secondary and Exploratory Outcomes. Secondary outcomes included 6MWD (27), LVEF (biplane Simpson method, blinded readers), HAMA (28) and HAMD (29) (Chinese versions, blinded assessors), and SF-36 (30) (Chinese version) with PCS and MCS scores. Safety outcomes included 12-week MACE, emergency-department visits, rehospitalization, and exercise-related adverse events.

### Statistical analysis

No formal *a priori* sample-size calculation was performed because the cohort size was determined by the number of eligible patients available during the prespecified study period. Accordingly, inference emphasized effect estimates and 95% confidence intervals. The study was not designed or powered to establish equivalence or non-inferiority between HBTCR and CBCR.

#### Confounding control

Generalized propensity scores were estimated via multinomial logistic regression in the 167 patients with clearly documented rehabilitation exposure, and overlap weights were applied to target the population with the greatest clinical equipoise. Twenty-five baseline covariates were included: age, sex, BMI, current smoking, hypertension, diabetes mellitus, number of grafts, baseline LVEF, baseline VO_2_peak, baseline 6MWD, digital literacy score, family support score, distance to the rehabilitation center, education, employment, urban residence, travel barrier score, schedule conflict score, Clinical Frailty Scale, NYHA class, postoperative atrial fibrillation, postoperative acute kidney injury, eGFR, HbA1c, and log-transformed NT-proBNP. Covariate balance was assessed using maximum absolute pairwise SMDs (threshold <0.10). Baseline distributions and balance diagnostics are summarized in Table 1; propensity-score overlap and Love plots are provided in Supplementary Figures S1, S2.

**Table 1:** Baseline characteristics before and after overlap weighting.

Characteristic	HBTCR (*n* = 56)	CBCR (*n* = 54)	Control (*n* = 57)	Max SMD (Before)	Max SMD (After)
Demographics					
Age, years	62.5 ± 8.1	63.6 ± 7.4	63.3 ± 8.1	0.152	0.019
Male, *n* (%)	45 (80.4)	46 (85.2)	46 (80.7)	0.127	0.007
BMI, kg/m²	26.8 ± 3.0	26.8 ± 3.2	27.3 ± 3.5	0.153	0.011
Cardiovascular risk factors					
Current smoker, *n* (%)	20 (35.7)	17 (31.5)	15 (26.3)	0.202	0.004
Hypertension, *n* (%)	40 (71.4)	41 (75.9)	39 (68.4)	0.167	0.034
Diabetes mellitus, *n* (%)	20 (35.7)	24 (44.4)	22 (38.6)	0.177	0.019
Postoperative AF, *n* (%)	8 (14.3)	8 (14.8)	9 (15.8)	0.042	0.015
Postoperative AKI, *n* (%)	6 (10.7)	11 (20.4)	7 (12.3)	0.266	0.003
eGFR, mL/min/1.73 m²	80.6 ± 21.1	72.0 ± 17.0	78.9 ± 15.3	0.452	0.029
HbA1c, %	6.2 ± 0.8	6.3 ± 0.9	6.1 ± 0.7	0.233	0.005
log NT-proBNP	5.2 ± 0.8	5.4 ± 0.8	5.3 ± 0.7	0.273	0.011
Surgical characteristics					
Number of grafts	3.1 ± 0.9	3.2 ± 0.8	3.1 ± 0.7	0.171	0.010
Baseline functional status					
LVEF, %	51.4 ± 5.5	50.5 ± 5.8	51.2 ± 4.8	0.162	0.008
Baseline VO_2_peak, mL/kg/min	18.7 ± 3.1	18.3 ± 3.1	18.1 ± 3.2	0.206	0.024
Baseline 6MWD, m	390.1 ± 63.7	383.3 ± 60.6	373.8 ± 66.1	0.250	0.015
Frailty (CFS)	1.9 ± 1.4	1.9 ± 1.4	2.5 ± 1.3	0.461	0.061
NYHA class	1.9 ± 0.8	2.2 ± 0.8	2.1 ± 0.7	0.293	0.040
Socioeconomic and access					
Digital literacy score	63.8 ± 12.7	57.4 ± 12.5	57.6 ± 15.4	0.497	0.014
Family support score	60.7 ± 13.3	61.7 ± 15.3	58.0 ± 14.4	0.244	0.006
Distance to center, km	6.8 ± 3.1	6.9 ± 3.1	7.4 ± 4.7	0.174	0.030
Education, years	12.1 ± 2.9	11.6 ± 2.6	11.2 ± 3.0	0.285	0.002
Employed, *n* (%)	33 (58.9)	24 (44.4)	15 (26.3)	0.692	0.021
Urban residence, *n* (%)	37 (66.1)	28 (51.9)	29 (50.9)	0.309	0.027
Travel barrier score	51.8 ± 9.0	51.9 ± 8.4	50.3 ± 9.5	0.173	0.013
Schedule conflict score	44.6 ± 12.9	43.8 ± 16.6	41.2 ± 13.4	0.259	0.010

Data are mean ± SD or *n* (%). The HBTCR, CBCR, and usual-care columns show unweighted observed data from the 167-patient resolved-exposure cohort. Maximum SMD is the largest absolute pairwise standardized mean difference before and after calibrated overlap weighting; missing baseline covariates were imputed before propensity-score estimation. All post-weighting SMDs were <0.10. BMI, body mass index; LVEF, left ventricular ejection fraction; VO_2_peak, peak oxygen consumption; 6MWD, 6-minute walk distance; NYHA, New York Heart Association; CFS, Clinical Frailty Scale; eGFR, estimated Glomerular Filtration Rate; AF, atrial fibrillation; AKI, acute kidney injury; NT-proBNP, N-terminal pro-B-type natriuretic peptide.

#### Primary and key outcome analyses

Adherence was compared using overlap-weighted linear regression with HC3 robust standard errors in the 110 HBTCR/CBCR patients with resolved exposure. Twelve-week VO_2_peak was analyzed using weighted ANCOVA with the 12-week value as the dependent variable and baseline VO_2_peak as a covariate; calibrated overlap weights were combined with inverse-probability-of-observation weights to address missing 12-week assessments. Comparisons with usual care were supportive. Secondary outcomes used the same weighted ANCOVA framework. Results are reported as adjusted mean differences with 95% CIs and exact *P* values (Table 2).

**Table 2:** Adjusted primary and secondary outcomes at 12 weeks.

Outcome	Group	Baseline	12 weeks	Adj. diff. vs. control (95% CI)	*P* (vs. control)	Adj. diff. HBTCR vs. CBCR (95% CI)	*P*	*N*
Primary implementation								
Adherence, %	HBTCR	–	87.7 ± 8.8	–	–	9.1 (4.4, 13.7)	<0.001	110
	CBCR	–	78.5 ± 10.8	–	–	(ref)	(ref)	110
Key clinical								
VO_2_peak, mL/kg/min	HBTCR	18.4 ± 3.0	23.6 ± 3.5	4.48 (3.72, 5.25)	<0.001	0.17 (−0.50, 0.84)	0.616	105
	CBCR	18.2 ± 3.2	23.1 ± 3.6	4.31 (3.60, 5.02)	<0.001	(ref)	(ref)	105
	Control	18.5 ± 2.9	19.1 ± 2.8	(ref)	(ref)	–	–	105
Secondary outcomes								
6MWD, m	HBTCR	381.5 ± 56.2	469.9 ± 61.6	75.3 (67.2, 83.4)	<0.001	−0.26 (−9.58, 9.07)	0.957	105
	CBCR	379.8 ± 59.1	463.9 ± 63.7	75.6 (68.3, 82.9)	<0.001	(ref)	(ref)	105
	Control	383.1 ± 55.7	393.6 ± 55.1	(ref)	(ref)	–	–	105
LVEF, %	HBTCR	51.4 ± 5.3	57.3 ± 5.7	5.24 (4.01, 6.47)	<0.001	0.93 (−0.40, 2.25)	0.171	105
	CBCR	51.1 ± 5.5	56.4 ± 5.6	4.31 (3.15, 5.48)	<0.001	(ref)	(ref)	105
	Control	51.6 ± 5.1	52.4 ± 5.4	(ref)	(ref)	–	–	105
HAMA	HBTCR	15.3 ± 4.2	7.0 ± 5.8	−6.61 (−7.71, −5.51)	<0.001	0.12 (−1.45, 1.70)	0.877	105
	CBCR	15.6 ± 4.4	7.7 ± 6.5	−6.73 (−8.09, −5.37)	<0.001	(ref)	(ref)	105
	Control	15.5 ± 4.0	14.4 ± 5.6	(ref)	(ref)	–	–	105
HAMD	HBTCR	16.9 ± 4.7	7.4 ± 6.2	−8.17 (−9.80, −6.54)	<0.001	−0.12 (−2.29, 2.06)	0.915	105
	CBCR	17.3 ± 4.9	8.2 ± 7.6	−8.05 (−9.67, −6.43)	<0.001	(ref)	(ref)	105
	Control	16.9 ± 4.3	15.4 ± 6.3	(ref)	(ref)	–	–	105
SF-36 QOL								
SF-36 PCS	HBTCR	53.0 ± 8.9	77.1 ± 12.7	21.7 (16.6, 26.8)	<0.001	2.20 (−2.59, 6.98)	0.368	105
	CBCR	51.7 ± 10.1	74.3 ± 13.6	19.5 (14.2, 24.8)	<0.001	(ref)	(ref)	105
	Control	53.6 ± 9.3	56.8 ± 9.5	(ref)	(ref)	–	–	105
SF-36 MCS	HBTCR	53.2 ± 10.4	73.5 ± 13.4	16.0 (12.1, 20.0)	<0.001	−2.24 (−6.78, 2.31)	0.335	105
	CBCR	51.3 ± 11.4	70.6 ± 14.1	18.2 (13.3, 23.2)	<0.001	(ref)	(ref)	105
	Control	51.9 ± 10.3	53.8 ± 14.6	(ref)	(ref)	–	–	105

Adherence was analyzed in the 110 HBTCR/CBCR patients with resolved exposure. Clinical outcomes were analyzed in the 105 complete cases using weighted ANCOVA with baseline adjustment and combined calibrated overlap and inverse-probability-of-observation weights. N indicates the analysis sample size for each outcome.

#### Multiplicity adjustment

A hierarchical testing strategy was prespecified: adherence, VO_2_peak, 6MWD. Holm correction was applied for key secondary outcomes; Benjamini–Hochberg FDR for SF-36 domains and other exploratory outcomes.

#### Missing data

Ninety of 195 potentially eligible patients (46.2%) were not included in the complete-case outcome analysis because of unresolved rehabilitation exposure (*n* = 28) or missing 12-week outcome assessments after exposure resolution (*n* = 62). Included and non-included patients were compared to characterize selection related to analysis inclusion (Table 3). Missing baseline covariates were addressed using multiple imputation by chained equations (MICE; *m* = 20) before generalized propensity-score estimation. For missing 12-week outcomes, the primary complete-case ANCOVA incorporated inverse-probability-of-observation weighting; a multiple-imputation sensitivity analysis was additionally performed in the 167-patient resolved-exposure cohort.

**Table 3:** Comparison of included and non-included patients.

Characteristic	Included (*n* = 105)	Non-included (*n* = 90)	SMD
Age, years	62.8 ± 8.5	63.6 ± 6.8	0.11
Male, *n* (%)	87 (82.9)	74 (82.2)	0.02
BMI, kg/m²	27.1 ± 3.4	27.0 ± 3.2	0.03
Diabetes mellitus, *n* (%)	39 (37.1)	38 (42.2)	0.10
Hypertension, *n* (%)	72 (68.6)	64 (71.1)	0.06
Current smoker, *n* (%)	33 (31.4)	29 (32.2)	0.02
Prior myocardial infarction, *n* (%)	42 (40.0)	32 (35.6)	0.09
Prior PCI, *n* (%)	33 (31.4)	38 (42.2)	0.23
LVEF, %	51.4 ± 5.3	50.9 ± 5.5	0.10
Baseline VO_2_peak, mL/kg/min	18.4 ± 3.0	18.3 ± 3.2	0.03
Baseline 6MWD, m	381.5 ± 56.5	380.8 ± 73.9	0.01
Urban residence, *n* (%)	61 (58.1)	51 (56.7)	0.03
Smartphone access, *n* (%)	82 (78.1)	72 (80.0)	0.05
Education, years	11.9 ± 2.9	10.9 ± 2.6	0.36
Education >12 years, *n* (%)	48 (45.7)	23 (25.6)	0.42
Employed, *n* (%)	51 (48.6)	29 (32.2)	0.34
Clinical Frailty Scale ≥4, *n* (%)	7 (6.7)	21 (23.3)	0.49
Family support score	64.5 ± 13.0	52.8 ± 13.0	0.89
Digital literacy score	59.9 ± 14.3	58.2 ± 14.2	0.12
Distance to center, km	6.9 ± 3.8	7.2 ± 3.7	0.08
Number of grafts	3.2 ± 0.8	3.1 ± 0.9	0.20

Data are mean ± SD or *n* (%). SMD, standardized mean difference. Of 195 potentially eligible patients, 90 (46.2%) were not included in the complete-case outcome analysis: 28 had unresolved/mixed rehabilitation exposure and 62 had missing 12-week outcomes after exposure resolution. LVEF, left ventricular ejection fraction; PCI, percutaneous coronary intervention.

#### Sensitivity analyses

Sensitivity analyses for the HBTCR–CBCR VO_2_peak comparison included unweighted complete-case ANCOVA, calibrated overlap weighting without observation weights, multiple imputation in the 167-patient resolved-exposure cohort, restriction to assessments obtained 11–13 weeks after the index date, and exclusion of modality crossovers (Supplementary Table S3). All analyses were performed using R version 4.3 and Python 3.11.

#### Exploratory adherence–outcome analysis

In the 70 HBTCR/CBCR participants with complete 12-week outcomes, a weighted multivariable linear model examined the association between objectively verified adherence and VO_2_peak change. The model included mean-centered adherence (per 10 percentage points), rehabilitation modality, an adherence × modality interaction, baseline VO_2_peak, age, sex, BMI, diabetes, education, family support, and digital literacy, and used the combined outcome weights with HC3 robust standard errors. This analysis was explicitly exploratory and was not interpreted causally.

## Results

### Patient selection and cohort characteristics

From 288 screened patients, 195 were potentially eligible. Of these, 90 patients (46.2%) were not included because of missing 12-week outcome assessments (*n* = 62) or unresolved rehabilitation exposure (*n* = 28). A comparison of baseline characteristics between included (*n* = 105) and non-included (*n* = 90) patients is presented in Table 3. Non-included patients had fewer years of education (10.9 ± 2.6 vs. 11.9 ± 2.9 years; SMD 0.36), were more often classified as frail (Clinical Frailty Scale ≥4: 23.3% vs. 6.7%), were less likely to be employed (32.2% vs. 48.6%), and reported lower family support scores (52.8 ± 13.0 vs. 64.5 ± 13.0; SMD 0.89); age, comorbidity burden, and baseline functional capacity were otherwise broadly comparable between included and non-included patients (all other SMDs ≤0.23). The missingness pattern is visualized in Supplementary Figure S3. The detailed flow is presented in Figure 2.

**Figure 2:**
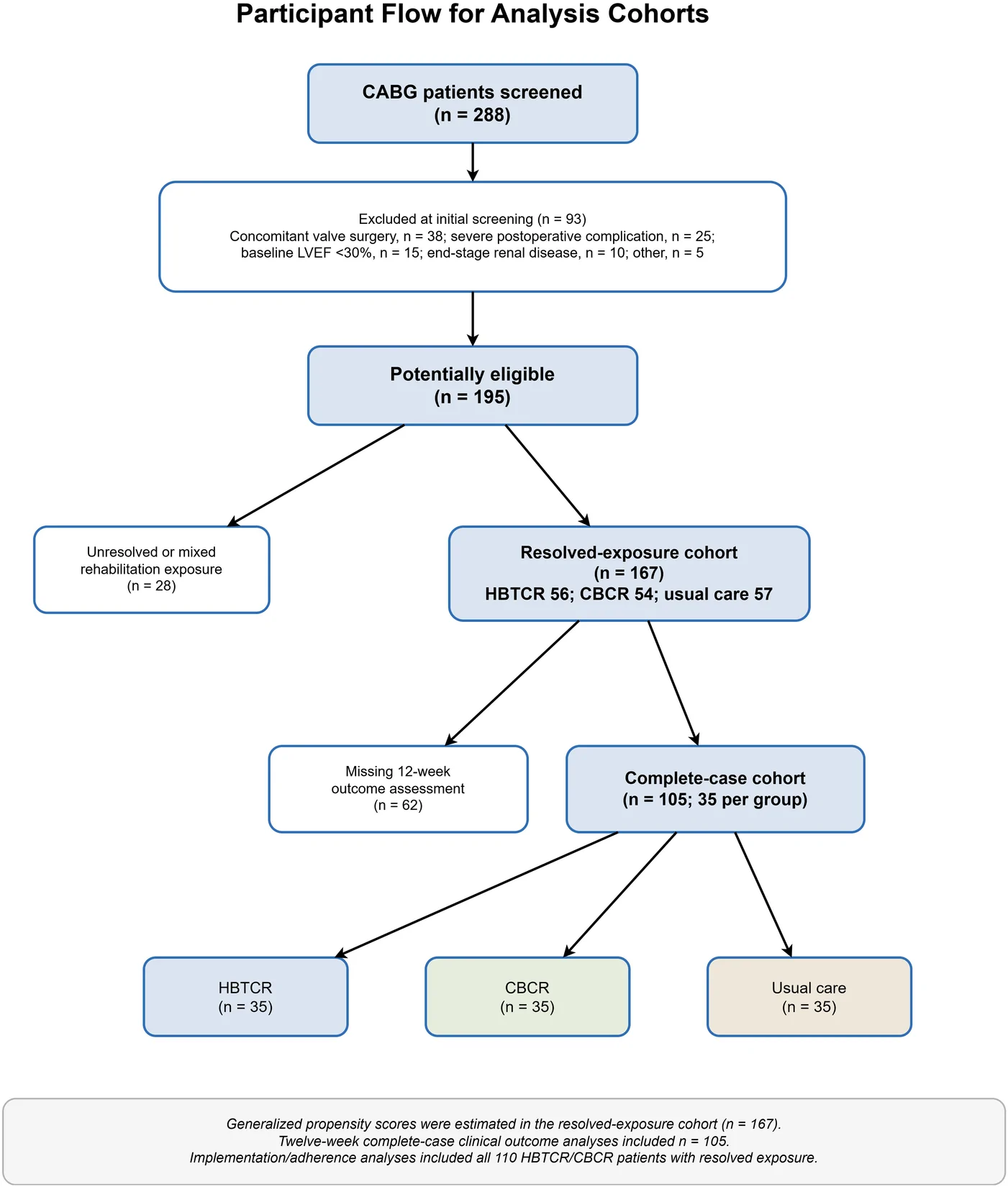
Participant flow diagram. Of 288 screened CABG patients, 93 were excluded at initial screening (concomitant valve surgery, *n* = 38; severe postoperative complication, *n* = 25; baseline LVEF <30%, *n* = 15; end-stage renal disease, *n* = 10; other exclusion, *n* = 5), leaving 195 potentially eligible patients. Rehabilitation exposure was unresolved or mixed in 28, leaving 167 patients with clear exposure for generalized propensity-score estimation (HBTCR *n* = 56, CBCR *n* = 54, usual care *n* = 57). Sixty-two lacked complete 12-week outcome assessment, leaving 105 complete cases (35 per group).

Baseline characteristics of the 167 patients with clearly documented exposure, together with covariate balance before and after overlap weighting, are presented in Table 1. Before weighting, patients who received HBTCR differed from those receiving CBCR in several practical dimensions: they were more likely to be employed (58.9% vs. 44.4%), had higher digital literacy scores (63.8 ± 12.7 vs. 57.4 ± 12.5; maximum pairwise SMD for digital literacy 0.50), and were more often urban residents (66.1% vs. 51.9%). Age, sex, baseline functional capacity, and distance to the rehabilitation center were broadly similar between HBTCR and CBCR. After calibrated overlap weighting, all maximum absolute pairwise SMDs were below 0.10, indicating satisfactory balance.

### Primary implementation outcome: session completion

Among the 110 rehabilitation patients in the exposure cohort, the mean objectively verified session completion was 87.7% (SD 8.8%) in HBTCR and 78.5% (SD 10.8%) in CBCR. The overlap-weighted between-group difference was 9.1 percentage points (95% CI: 4.4 to 13.7; *P* < 0.001). The proportion achieving ≥80% completion was 78.6% (44/56) in HBTCR versus 44.4% (24/54) in CBCR. Detailed session-level data are presented in Table 4 and the adherence distribution is shown in Figure 3. Technical failures affected 1.7% of HBTCR sessions.

**Table 4:** Session completion and training dose.

Measure	HBTCR (*n* = 56)	CBCR (*n* = 54)	Adjusted difference (95% CI)	*P*
Prescribed sessions	2,016	1,944	–	–
Completed sessions, mean ± SD	31.6 ± 3.1	28.3 ± 3.8	3.3 (1.7, 4.9)	<0.001
Completion rate, %, mean ± SD	87.7 ± 8.8	78.5 ± 10.8	9.1 (4.4, 13.7)	<0.001
≥80% completion, *n* (%)	44 (78.6)	24 (44.4)	–	<0.001
≥90% completion, *n* (%)	26 (46.4)	7 (13.0)	–	<0.001
Objectively verified completed exercise minutes, mean ± SD	1,578 ± 159	1,413 ± 195	163 (80, 247)	<0.001
Target-intensity exercise minutes, mean ± SD	1,230 ± 145	1,095 ± 154	124 (53, 194)	<0.001
Scheduled tele-consultations, mean ± SD	10.5 ± 1.2	–	–	–
Recorded technical-failure episodes, *n* (% of prescribed sessions)	34 (1.7)	0 (0)	–	–

A session was considered completed when ≥30 min of prescribed exercise at target intensity were objectively documented. Adjusted continuous differences were estimated using calibrated overlap-weighted linear regression with HC3 robust standard errors. Threshold *P* values are from Fisher exact tests. HBTCR, home-based telerehabilitation; CBCR, center-based cardiac rehabilitation.

**Figure 3:**
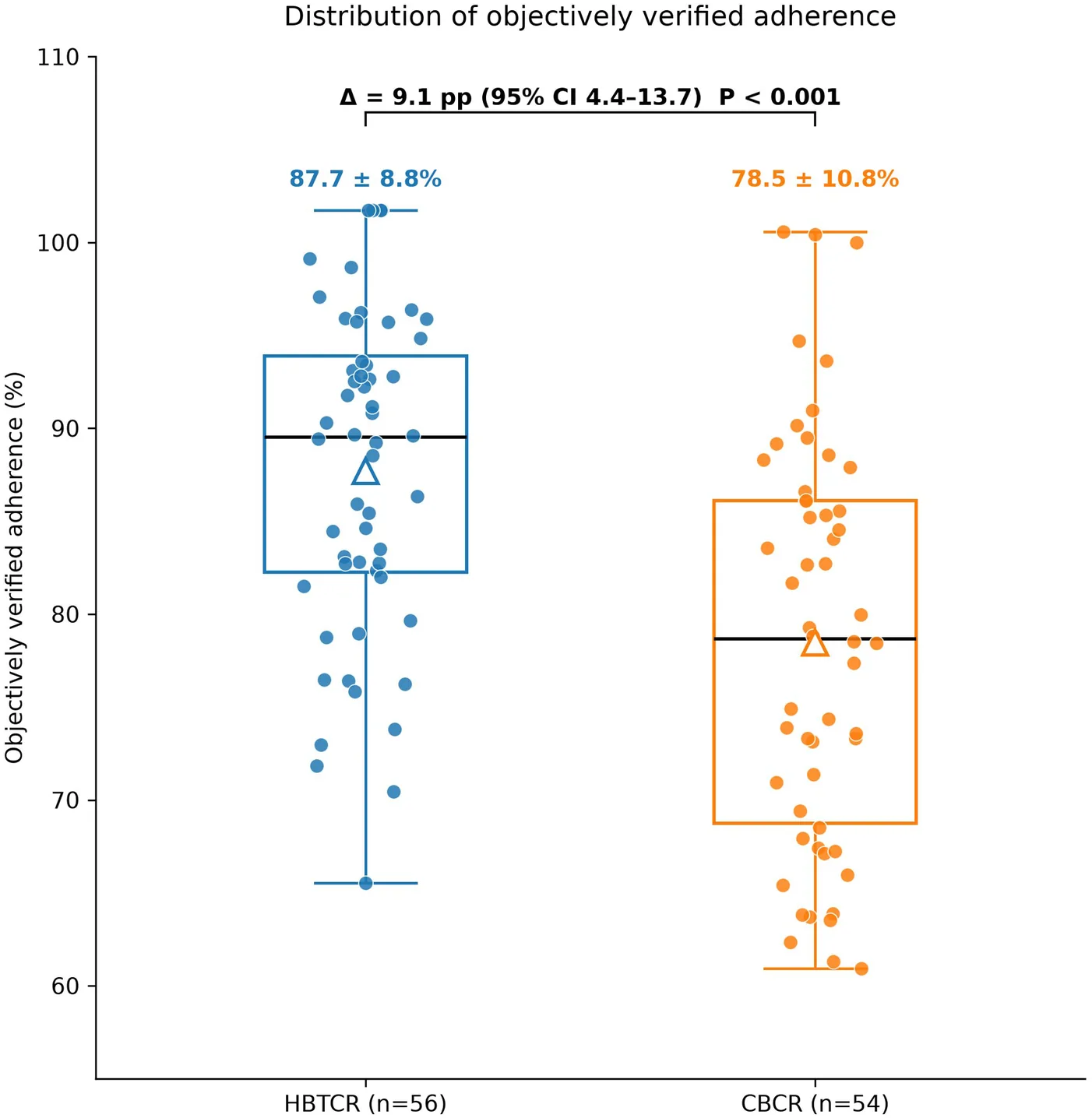
Session completion by rehabilitation modality. Raincloud plot showing individual data points, box plots (median, IQR, whiskers), and density distributions of session completion percentages in HBTCR and CBCR groups (*n* = 110). Mean values: HBTCR 87.7% (SD 8.8%), CBCR 78.5% (SD 10.8%). Overlap-weighted difference: 9.1 percentage points (95% CI: 4.4–13.7; *P* < 0.001).

### Key clinical outcome and secondary endpoints

The adjusted mean difference in 12-week VO_2_peak between HBTCR and CBCR was 0.17 mL/kg/min (95% CI: −0.50 to 0.84; *P* = 0.616). Both active groups showed substantially larger adjusted values than usual care (HBTCR vs. control: +4.48 mL/kg/min, 95% CI: 3.72 to 5.25; CBCR vs. control: +4.31 mL/kg/min, 95% CI: 3.60 to 5.02).

Secondary outcomes showed similar patterns (Table 2, Figures 4–6). Adjusted HBTCR–CBCR differences were: 6MWD −0.26 m (95% CI: −9.58 to 9.07; *P* = 0.957), LVEF 0.93% (95% CI: −0.40 to 2.25; *P* = 0.171), HAMA 0.12 (95% CI: −1.45 to 1.70; *P* = 0.877), HAMD −0.12 (95% CI: −2.29 to 2.06; *P* = 0.915). SF-36 domain differences between HBTCR and CBCR were small and non-significant after FDR correction. All eight SF-36 domain estimates are reported in Supplementary Table S4.

**Figure 4:**
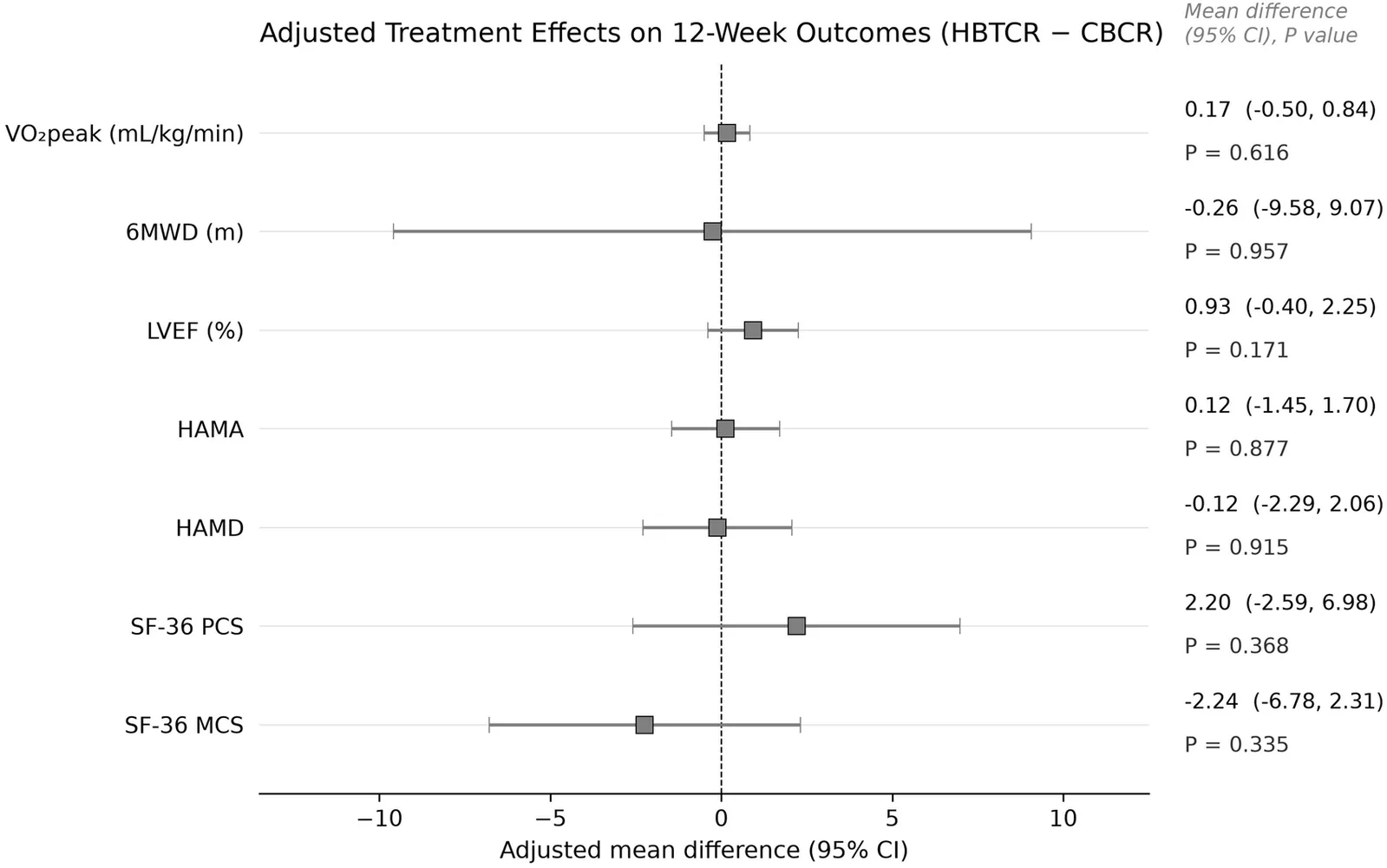
Adjusted treatment effects on functional outcomes. Forest plot of adjusted mean differences for VO_2_peak and 6MWD with 95% CIs, comparing HBTCR versus CBCR, HBTCR versus usual care, and CBCR versus usual care. Estimates are from weighted ANCOVA with baseline adjustment.

**Figure 5:**
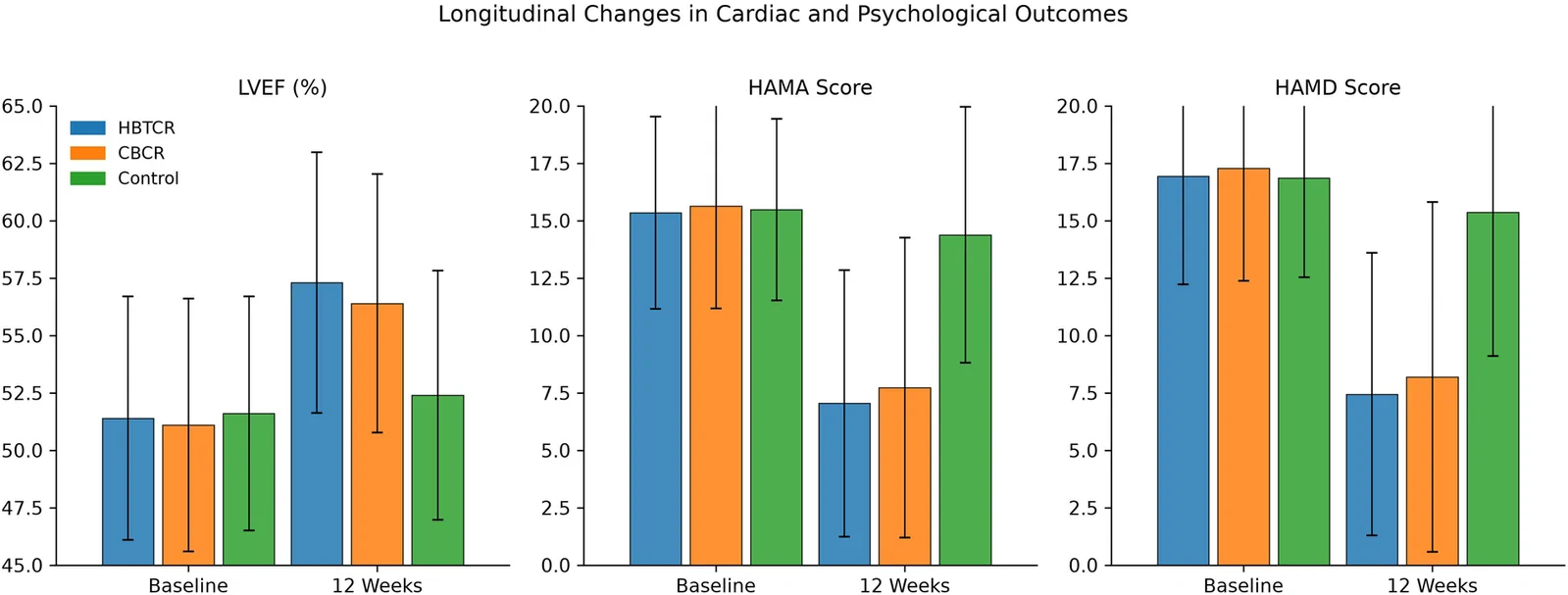
Longitudinal changes in cardiac and psychological outcomes. Three-panel figure: **(A)** LVEF trajectory, **(B)** HAMA score trajectory, **(C)** HAMD score trajectory from baseline to 12 weeks by group (HBTCR, CBCR, usual care). Error bars represent standard errors. *P*-values from weighted ANCOVA.

**Figure 6:**
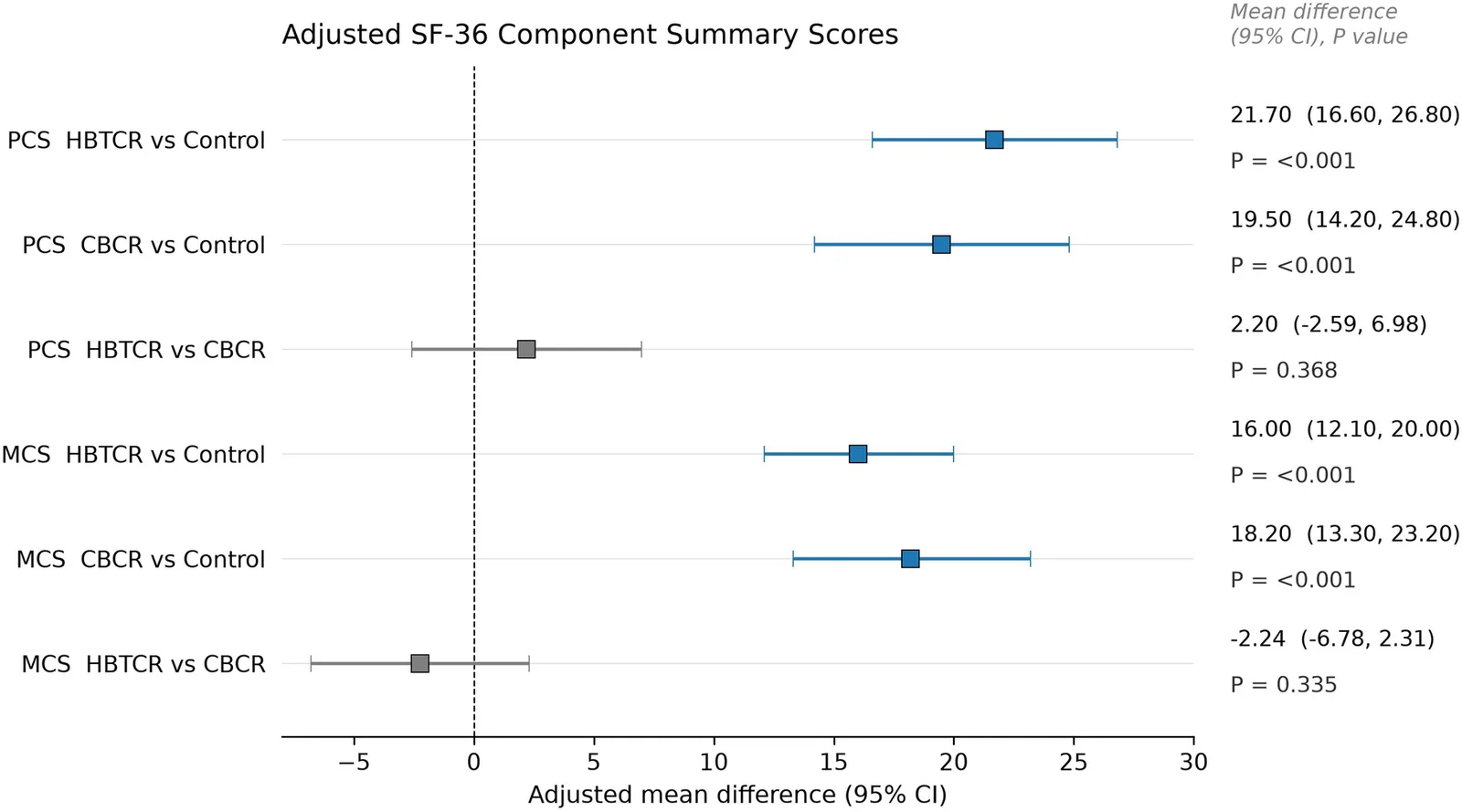
SF-36 component summary scores. Adjusted mean differences in PCS and MCS comparing HBTCR and CBCR versus usual care, with 95% CIs. Non-significant after Benjamini–Hochberg FDR correction.

### Safety outcomes

No 12-week MACE occurred in HBTCR or CBCR; two MACE occurred in usual care (2/35, 5.7%). Emergency-department visits occurred in 3/35 (8.6%) HBTCR, 1/35 (2.9%) CBCR, and 2/35 (5.7%) usual-care patients; rehospitalization occurred in 2/35 (5.7%), 1/35 (2.9%), and 3/35 (8.6%), respectively. At least one exercise-related adverse event was recorded in 6/35 (17.1%) HBTCR and 4/35 (11.4%) CBCR participants; because event counts were small and usual-care patients were not enrolled in a formal exercise program, no formal comparative safety inference was made. Technical failures comprised 34 recorded episodes across 2,016 prescribed HBTCR sessions (1.7%) (Table 5; Supplementary Table S5).

**Table 5:** Safety outcomes.

Event	HBTCR (*n* = 35)	CBCR (*n* = 35)	Usual care (*n* = 35)
12-week MACE	0	0	2 (5.7)
Emergency-department visit	3 (8.6)	1 (2.9)	2 (5.7)
Rehospitalization	2 (5.7)	1 (2.9)	3 (8.6)
Any exercise-related adverse event	6 (17.1)	4 (11.4)	–
Exercise-related chest pain	2 (5.7)	2 (5.7)	–
Exercise-related arrhythmia	1 (2.9)	1 (2.9)	–
Symptomatic hypotension	1 (2.9)	0	–
Fall during exercise	1 (2.9)	0	–
Musculoskeletal injury	2 (5.7)	1 (2.9)	–

Values are *n* (%). MACE, major adverse cardiovascular events. Exercise-related adverse events are shown only for participants enrolled in formal rehabilitation because usual-care patients did not participate in a prescribed rehabilitation exercise program. Event counts were small; no formal between-group safety testing was performed.

### Sensitivity analyses

The primary HBTCR–CBCR VO_2_peak estimate was directionally consistent across sensitivity analyses: unweighted complete-case ANCOVA, 0.29 mL/kg/min (95% CI: −0.30 to 0.89; *P* = 0.334); calibrated overlap weighting alone, 0.23 (−0.40 to 0.86; *P* = 0.477); multiple imputation, 0.39 (−1.32 to 2.11; *P* = 0.643; *n* = 167); restriction to the 11–13-week window, 0.001 (−0.97 to 0.97; *P* = 0.999; *n* = 63); and exclusion of modality crossovers, 0.16 (−0.52 to 0.83; *P* = 0.644; *n* = 103) (Table 6; Supplementary Table S3).

**Table 6:** Sensitivity analyses for VO_2_peak.

Analysis	Adjusted mean difference (mL/kg/min)	95% CI	*P*	*N*
Primary (calibrated overlap + IPOW)	0.17	−0.50 to 0.84	0.616	105
Unweighted complete-case ANCOVA	0.29	−0.30 to 0.89	0.334	105
Calibrated overlap weights	0.23	−0.40 to 0.86	0.477	105
Multiple imputation (*m* = 20)	0.39	−1.32 to 2.11	0.643	167
Strict 11–13-week window	0.001	−0.97 to 0.97	0.999	63
Excluding modality crossovers	0.16	−0.52 to 0.83	0.644	103

All analyses compare HBTCR with CBCR for 12-week VO_2_peak. The primary model used calibrated overlap weights combined with inverse-probability-of-observation weighting (IPOW). MICE = Multiple Imputation by Chained Equations (*m* = 20).

### Exploratory analysis: adherence and functional improvement

In the 70 rehabilitation participants with complete 12-week outcomes, the weighted adjusted association between adherence and VO_2_peak improvement was positive but did not reach conventional statistical significance (*β* = 0.62 mL/kg/min per 10-percentage-point increase in adherence; 95% CI: −0.03 to 1.27; *P* = 0.062). The adherence × HBTCR interaction was also non-significant (*β* = 0.36; 95% CI: −0.42 to 1.14; *P* = 0.368). These estimates (Figure 7; Table 7; Supplementary Table S6) are exploratory and do not establish a causal dose–response relationship; healthier, more motivated, or faster-recovering patients may both complete more sessions and improve more.

**Figure 7:**
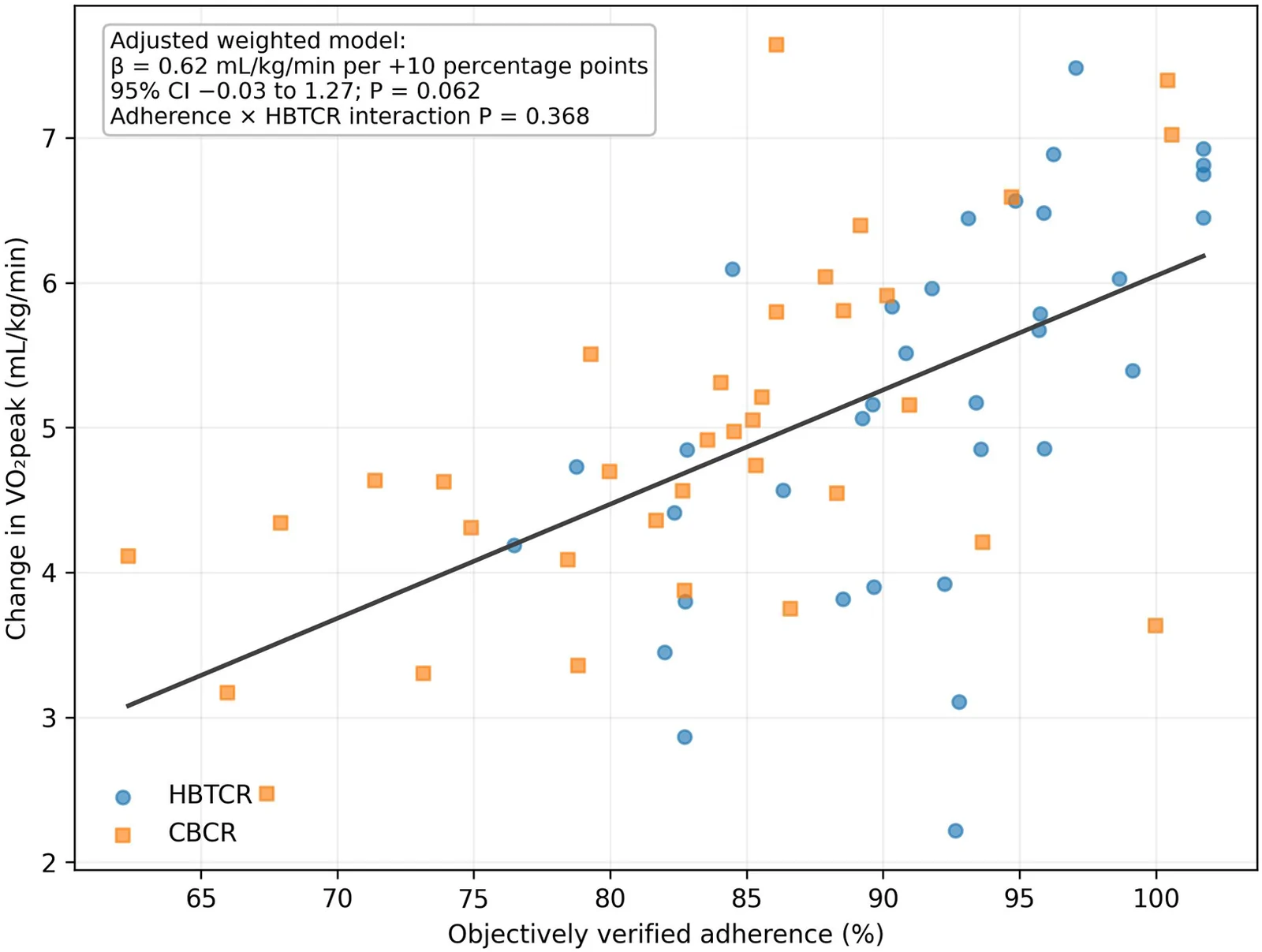
Exploratory adherence–VO_2_peak association. Scatter plot showing the observed relationship between objectively verified adherence (%) and change in VO_2_peak (mL/kg/min) among rehabilitation participants with complete 12-week outcomes (*n* = 70). In the weighted multivariable model, the adjusted association was *β* = 0.62 mL/kg/min per 10-percentage-point increase in adherence (95% CI: −0.03 to 1.27; *P* = 0.062), and the adherence × HBTCR interaction was not statistically significant (*P* = 0.368). The fitted line is descriptive; the adjusted model estimates are reported in the annotation. This exploratory analysis does not establish causality.

**Table 7:** Exploratory adherence–VO_2_peak association.

Variable	*β* Coefficient	95% CI	*P*
Adherence (per 10 percentage points)	0.62	−0.03, 1.27	0.062
HBTCR vs. CBCR at mean adherence	−0.35	−1.10, 0.39	0.354
Adherence × HBTCR interaction	0.36	−0.42, 1.14	0.368
Baseline VO_2_peak (per 1 mL/kg/min)	0.02	−0.19, 0.24	0.834
Age (per 10 years)	0.03	−0.61, 0.67	0.933
Male vs. female	0.06	−1.17, 1.29	0.922
BMI (per 5 kg/m²)	0.08	−0.52, 0.68	0.792
Diabetes (yes vs. no)	−0.22	−0.83, 0.39	0.471
Education (per year)	0.01	−0.15, 0.17	0.899
Family support (per 10 points)	−0.16	−0.40, 0.08	0.193
Digital literacy (per 10 points)	−0.12	−0.41, 0.16	0.394

*N* = 70 rehabilitation participants with complete 12-week outcomes. Weighted multivariable linear regression used combined outcome weights and HC3 robust standard errors. Adherence was mean-centered at 87.35% for the modality main effect and scaled per 10 percentage points. Model *R*^2^ = 0.399; adjusted *R*^2^ = 0.285. This exploratory analysis does not establish causality.

## Discussion

In this single-center observational cohort study of post-CABG patients, HBTCR was associated with higher objectively verified session completion than CBCR (overlap-weighted difference 9.1 percentage points; 95% CI: 4.4 to 13.7). The adjusted difference in 12-week VO_2_peak was small (0.17 mL/kg/min; 95% CI: −0.50 to 0.84) and not statistically significant. The confidence interval spans a small advantage for CBCR to a modest advantage for HBTCR and therefore does not establish equivalence or non-inferiority. Both active rehabilitation models were associated with substantially larger 12-week functional improvements than usual care.

The higher observed adherence in HBTCR (87.7% vs. 78.5%) merits careful interpretation. While this may reflect genuine advantages of home-based delivery (elimination of travel, flexible scheduling (12, 15)), alternative explanations must be considered: residual differential ascertainment between device-verified and directly observed sessions; systematic differences between patients choosing each modality (digital literacy, employment, motivation); and the exclusion of 46.2% of potentially eligible patients, who had lower educational attainment, more frailty, lower employment, and less family support than included patients. These factors may produce a “healthy adherer” effect that cannot be fully resolved in an observational design (31, 32).

The exploratory adherence–outcome association (*β* = 0.62 mL/kg/min per 10-percentage-point increase; 95% CI: −0.03 to 1.27) did not reach conventional statistical significance after adjustment and must not be interpreted causally. The association may be confounded by factors influencing both adherence and recovery, including baseline health status, motivation, digital capability, and social support. Separately, the reductions in anxiety and depressive symptoms observed with active rehabilitation are consistent with prior reports emphasizing the burden and prognostic relevance of psychological symptoms after CABG and in cardiovascular disease (33–35).

Recorded exercise-related events were uncommon in absolute numbers but were not absent, and the study was too small for reliable between-program safety comparisons. Two 12-week MACE occurred in the usual-care group, whereas none occurred in either active rehabilitation group. Technical-failure frequency was low (34 recorded episodes, 1.7 per 100 prescribed HBTCR sessions). These observations support operational feasibility in selected patients but should not be interpreted as comparative safety evidence.

This study has several limitations. First, as an observational study using routinely collected data, we cannot establish causality; unmeasured confounding from rehabilitation preference and motivation may persist. Second, the exclusion of 46.2% of potentially eligible patients raises concerns about selection bias, although sensitivity analyses were consistent with the primary analysis. Third, this was a single-center study with 12-week follow-up, limiting generalizability and precluding assessment of long-term outcomes. Fourth, the sample size was fixed by the available cohort and was insufficient for formal equivalence or non-inferiority testing; therefore, the HBTCR–CBCR effect estimate and its 95% confidence interval should be interpreted as measures of statistical precision rather than evidence of comparable effectiveness. Fifth, despite efforts to harmonize adherence definitions, residual differential measurement may persist; in particular, the two delivery models differed in supervision intensity, and adherence ascertainment relied on device verification at home versus direct observation in center. Finally, although we verified that no patient participated in both the present cohort and our previously reported trial, the two studies share the same institutional practice environment, and these findings should be interpreted as complementary rather than fully independent evidence.

## Conclusion

In this single-center observational analysis of post-CABG patients, those who chose HBTCR achieved higher objectively verified session completion and comparable observed 12-week improvement in VO_2_peak to those who chose CBCR. Both active rehabilitation models were associated with substantially larger 12-week functional improvements than usual care. These findings support offering HBTCR as a feasible alternative for patients who prefer or require home-based rehabilitation, including those for whom work, travel, or scheduling constraints may limit attendance at center-based programs. The study was not designed for formal equivalence testing; the clinical meaningfulness and cost-effectiveness of HBTCR should be evaluated in future studies.

## Related publications and participant overlap

A prior randomized trial conducted at our center evaluated home-based versus center-based rehabilitation in a separate cohort of post-CABG patients recruited during 2019–2020 (20). Cross-checking of surgical dates and enrollment records confirmed that no participant was shared between that trial and the present 2024–2025 cohort. Novel versus previously reported variables are disclosed in Supplementary Table S1.

## Data Availability

The original contributions presented in the study are included in the article/Supplementary Material, further inquiries can be directed to the corresponding author.

## Supplementary material

The Supplementary Material for this article can be found online at: https://www.frontiersin.org/articles/10.3389/fcvm.2026.1947232/full#supplementary-material

SUPPLEMENTARY FIGURE S1 Generalized Propensity Score Overlap. Density distributions of estimated generalized propensity scores for HBTCR, CBCR, and usual care groups, demonstrating adequate overlap across the three treatment levels.

SUPPLEMENTARY FIGURE S2 Covariate Balance Love Plot. Maximum pairwise absolute standardized mean differences (SMDs) before and after calibrated overlap weighting. Dashed line indicates the SMD = 0.10 threshold. All covariates achieved post-weighting SMD < 0.10.

SUPPLEMENTARY FIGURE S3 Missingness Pattern. Matrix visualization of missing data patterns across covariates and outcome variables in the screened population (n=288); the 167-patient resolved-exposure cohort was used for propensity-score estimation and missing-outcome sensitivity analyses.
