# Supplementary material for: Home-based telerehabilitation after CABG: higher objectively verified adherence and 12-week functional outcomes in a single-center observational cohort
Source: Front Cardiovasc Med. 2026 Oct 15;13:1947232. doi: 10.3389/fcvm.2026.1947232 (PMC13716574; doi:10.3389/fcvm.2026.1947232)
Supplement: Supplementary file 1 [file Datasheet1.pdf]

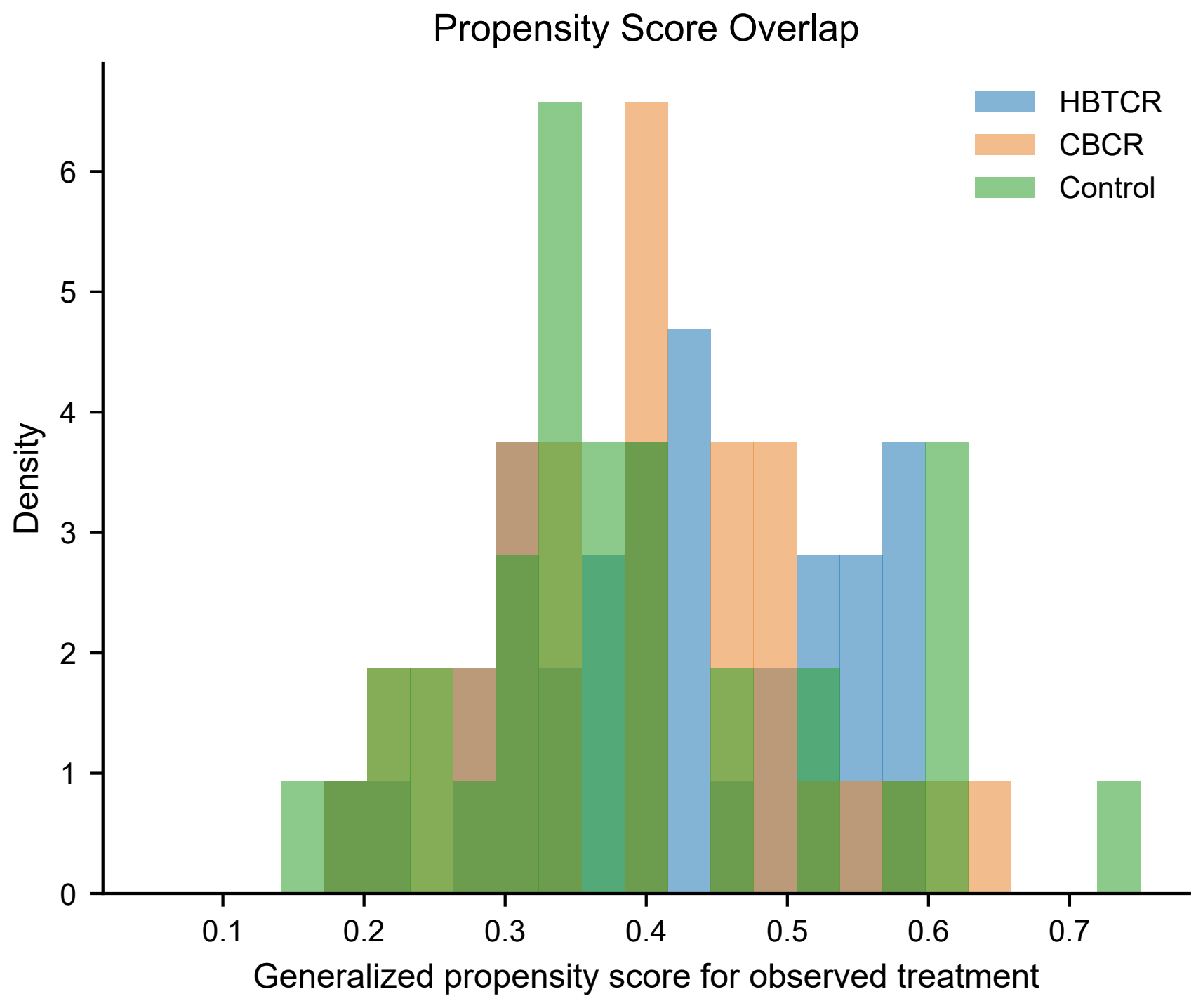

Propensity Score Overlap
HBTCR
CBCR
Control
Density
0
1
2
3
4
5
6
0.1
0.2
0.3
0.4
0.5
0.6
0.7
Generalized propensity score for observed treatment
