# Supplementary material for: Home-based telerehabilitation after CABG: higher objectively verified adherence and 12-week functional outcomes in a single-center observational cohort
Source: Front Cardiovasc Med. 2026 Oct 15;13:1947232. doi: 10.3389/fcvm.2026.1947232 (PMC13716574; doi:10.3389/fcvm.2026.1947232)
Supplement: Supplementary file 2 [file Datasheet2.pdf]

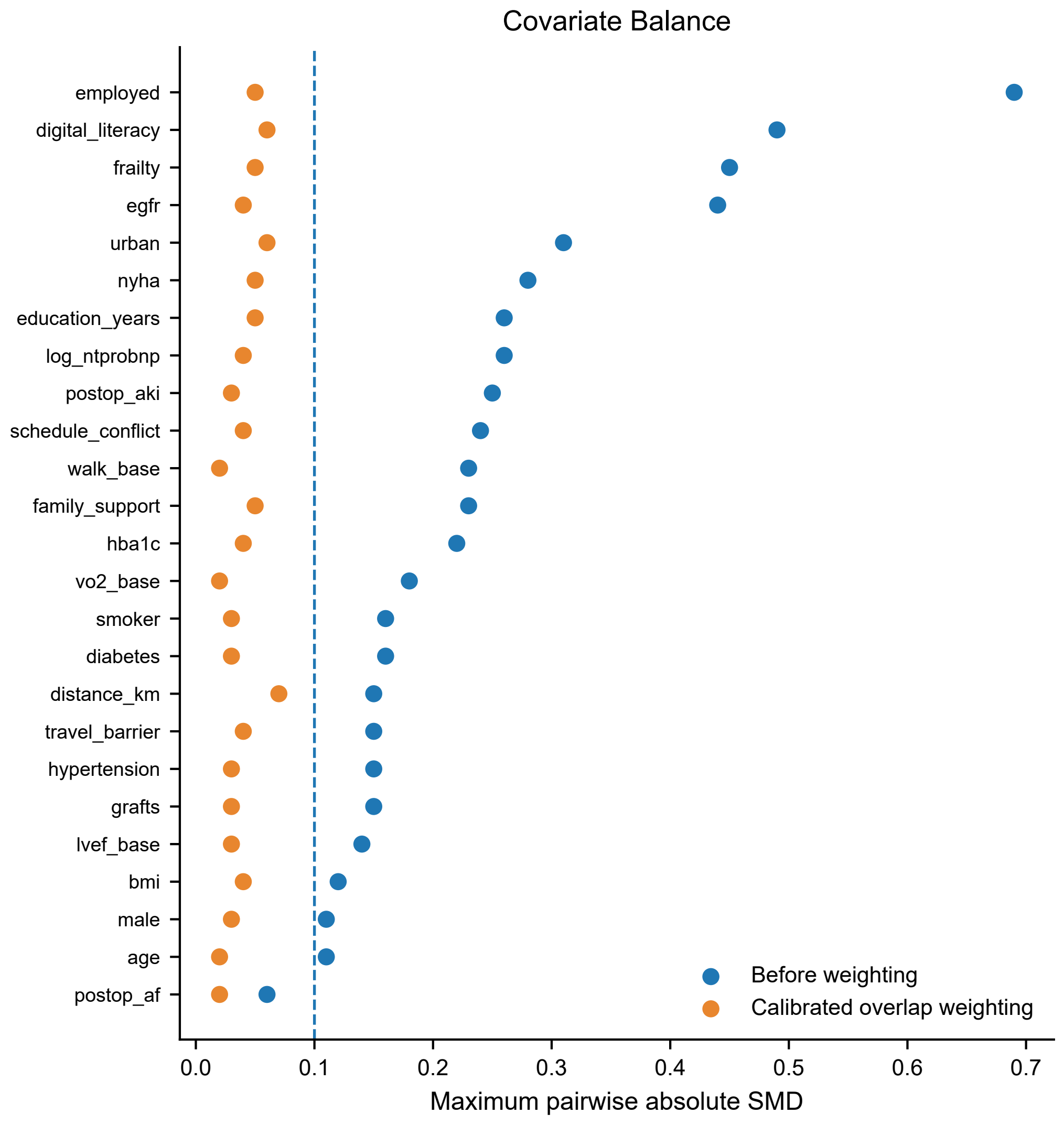

Covariate Balance
employed
digital_literacy
frailty
egfr
urban
nyha
education_years
log_ntprobnp
postop_aki
schedule_conflict
walk_base
family_support
hba1c
vo2_base
smoker
diabetes
distance_km
travel_barrier
hypertension
grafts
lvef_base
bmi
male
age
postop_af
0.0
0.1
0.2
0.3
0.4
0.5
0.6
0.7
Maximum pairwise absolute SMD
Before weighting
Calibrated overlap weighting
