# Supplementary material for: Home-based telerehabilitation after CABG: higher objectively verified adherence and 12-week functional outcomes in a single-center observational cohort
Source: Front Cardiovasc Med. 2026 Oct 15;13:1947232. doi: 10.3389/fcvm.2026.1947232 (PMC13716574; doi:10.3389/fcvm.2026.1947232)
Supplement: Supplementary file 3 [file Datasheet3.pdf]

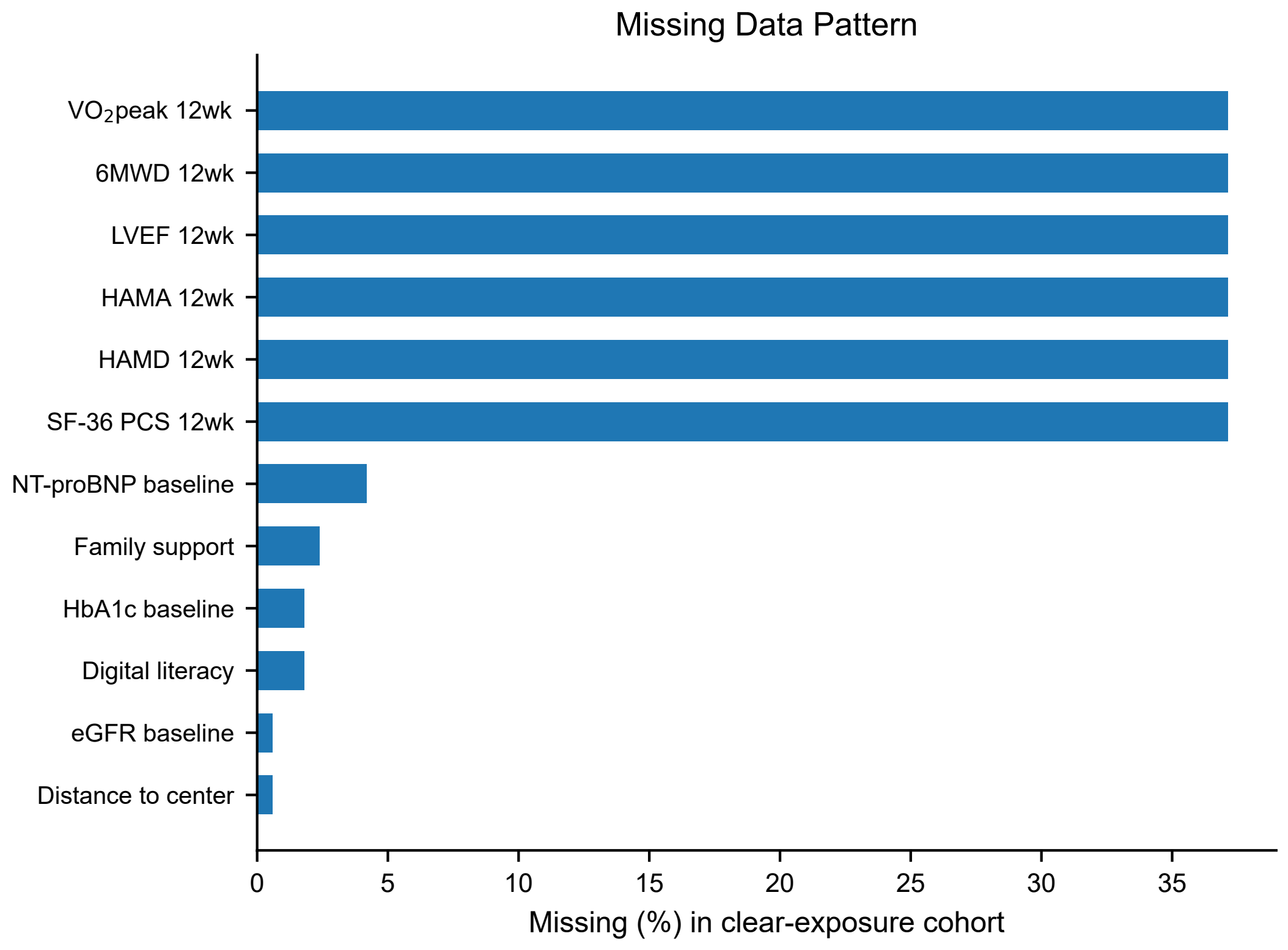
Missing Data Pattern
VO$_2$peak 12wk
6MWD 12wk
LVEF 12wk
HAMA 12wk
HAMD 12wk
SF-36 PCS 12wk
NT-proBNP baseline
Family support
HbA1c baseline
Digital literacy
eGFR baseline
Distance to center
0
5
10
15
20
25
30
35
Missing (%) in clear-exposure cohort
