# Supplementary material for: Home-based telerehabilitation after CABG: higher objectively verified adherence and 12-week functional outcomes in a single-center observational cohort
Source: Front Cardiovasc Med. 2026 Oct 15;13:1947232. doi: 10.3389/fcvm.2026.1947232 (PMC13716574; doi:10.3389/fcvm.2026.1947232)
Supplement: Supplementary file 4 [file Supplementaryfile1.docx]

**Supplementary Tables**

## Supplementary Table S1. Participant Overlap, Previously Reported Variables, and Analyses Unique to the Present Report

| **Domain** | **Previously Reported (Ma et al., 2021)** | **Present Report (Novel or Expanded)** |
| --- | --- | --- |
| Study design | Single-center RCT | Observational cohort using routinely collected EHR |
| Primary outcome | Psychological status (HAMA, HAMD) and QOL (SF-36) | Objectively verified session completion (adherence) |
| Key clinical outcome | Not reported | 12-week VO₂peak (mL/kg/min) via CPET |
| Adherence measurement | Self-reported session logs | Objectively verified wearable HR data (HBTCR) or attendance/telemetry records (CBCR) |
| Confounding control | Randomization | Generalized propensity scores with calibrated overlap weighting |
| Covariates adjusted | Age, sex, BMI, LVEF | 25 baseline covariates, including digital literacy, family support, distance, employment, frailty, and clinical variables |
| Missing data handling | Complete-case analysis | MICE (m=20) for baseline covariates; inverse-probability-of-observation weighting for missing 12-week outcomes |
| Sensitivity analyses | Not performed | Unweighted, overlap-only, multiple-imputation, strict-window, and crossover-exclusion analyses |
| Exploratory analysis | Not performed | Weighted adherence–VO₂peak association with modality interaction; explicitly non-causal |
| Safety reporting | Adverse events listed | Participant-level 12-week safety events and HBTCR technical-failure frequency |
| Reporting standard | CONSORT | STROBE + RECORD + TIDieR |
| Participant overlap | N=60 (HBTCR=30, CBCR=30), recruited 2019–2020 | Present cohort recruited February 2024–February 2025; enrollment cross-check confirmed no shared participants (0/60) |

HBTCR: Home-Based Telerehabilitation; CBCR: Center-Based Cardiac Rehabilitation; CPET: Cardiopulmonary Exercise Test; EHR: Electronic Health Records; IPOW: Inverse-Probability-of-Observation Weighting; MICE: Multiple Imputation by Chained Equations; RCT: Randomized Controlled Trial; VO₂peak: Peak Oxygen Consumption. Cross-checking of surgical dates and rehabilitation enrollment records against the prior trial log confirmed that no participant was shared between the present cohort and the previously reported trial by Ma et al. (2021).

## Supplementary Table S2. Detailed Session-Level Adherence and Training Dose Data

| **Measure** | **HBTCR (n=56)** | **CBCR (n=54)** | **Adjusted Difference (95% CI)** | **P Value** |
| --- | --- | --- | --- | --- |
| Prescribed sessions | 2,016 | 1,944 | – | – |
| Completed sessions, mean ± SD | 31.6 ± 3.1 | 28.3 ± 3.8 | 3.3 (1.7, 4.9) | <0.001 |
| Completion rate, %, mean ± SD | 87.7 ± 8.8 | 78.5 ± 10.8 | 9.1 (4.4, 13.7) | <0.001 |
| Completion rate, median (IQR) | 89.5 (82.2, 93.9) | 78.7 (68.7, 86.1) | – | – |
| ≥80% completion, n (%) | 44 (78.6) | 24 (44.4) | – | <0.001 |
| ≥90% completion, n (%) | 26 (46.4) | 7 (13.0) | – | <0.001 |
| Objectively verified completed exercise minutes, mean ± SD | 1,578 ± 159 | 1,413 ± 195 | 163 (80, 247) | <0.001 |
| Target-intensity exercise minutes, mean ± SD | 1,230 ± 145 | 1,095 ± 154 | 124 (53, 194) | <0.001 |
| Scheduled tele-consultations, mean ± SD | 10.5 ± 1.2 | – | – | – |
| Recorded technical-failure episodes, n | 34 | 0 | – | – |
| Technical-failure rate, % of prescribed sessions | 1.7 | 0 | – | – |
| Modality crossover, n (%) | 3 (5.4) | 4 (7.4) | – | – |

HBTCR: Home-Based Telerehabilitation; CBCR: Center-Based Cardiac Rehabilitation; IQR: Interquartile Range; SD: Standard Deviation. A session was considered completed when ≥30 minutes of prescribed exercise at target intensity were objectively documented. Continuous adjusted differences were estimated using calibrated overlap-weighted linear regression with HC3 robust standard errors; threshold P values are from Fisher exact tests.

## Supplementary Table S3. Sensitivity Analyses for VO₂peak (HBTCR vs. CBCR)

| **Analysis** | **Adjusted Mean Difference (mL/kg/min)** | **95% CI** | **P Value** | **N** |  |
| --- | --- | --- | --- | --- | --- |
| Primary (calibrated overlap + IPOW) | 0.17 | −0.50 to 0.84 | 0.616 | 105 |  |
| Unweighted complete-case ANCOVA | 0.29 | −0.30 to 0.89 | 0.334 | 105 |  |
| Calibrated overlap weights | 0.23 | −0.40 to 0.86 | 0.477 | 105 |  |
| Multiple imputation (m=20) | 0.39 | −1.32 to 2.11 | 0.643 | 167 |  |
| Strict 11–13-week window | 0.001 | −0.97 to 0.97 | 0.999 | 63 |  |
| Excluding modality crossovers | 0.16 | −0.52 to 0.83 | 0.644 | 103 |  |

MICE: Multiple Imputation by Chained Equations; IPOW: Inverse-Probability-of-Observation Weighting. All analyses compare HBTCR with CBCR for 12-week VO₂peak using baseline VO₂peak as a covariate.

## Supplementary Table S4. SF-36 Health Survey Domain Scores: Adjusted Between-Group Differences at 12 Weeks

| **SF-36 Domain** | **Contrast** | **Adjusted Difference** | **95% CI** | ***P* Value** | **FDR-adjusted P** |
| --- | --- | --- | --- | --- | --- |
| **Physical Component Summary** | HBTCR vs Control | 21.7 | 16.6, 26.8 | – | – |
| **Physical Component Summary** | CBCR vs Control | 19.5 | 14.2, 24.8 | – | – |
| **Physical Component Summary** | HBTCR vs CBCR | 2.20 | −2.59, 6.98 | 0.368 | – |
| **Mental Component Summary** | HBTCR vs Control | 16.0 | 12.1, 20.0 | – | – |
| **Mental Component Summary** | CBCR vs Control | 18.2 | 13.3, 23.2 | – | – |
| **Mental Component Summary** | HBTCR vs CBCR | −2.24 | −6.78, 2.31 | 0.335 | – |
| **Physical Functioning** | HBTCR vs Control | 21.91 | 15.89, 27.94 | < 0.001 | – |
| **Physical Functioning** | CBCR vs Control | 17.75 | 11.32, 24.18 | < 0.001 | – |
| **Physical Functioning** | HBTCR vs CBCR | 4.16 | -0.98, 9.31 | 0.113 | 0.226 |
| **Role-Physical** | HBTCR vs Control | 19.79 | 13.33, 26.25 | < 0.001 | – |
| **Role-Physical** | CBCR vs Control | 15.19 | 8.46, 21.91 | < 0.001 | – |
| **Role-Physical** | HBTCR vs CBCR | 4.60 | -0.95, 10.16 | 0.104 | 0.226 |
| **Bodily Pain** | HBTCR vs Control | 21.40 | 14.47, 28.34 | < 0.001 | – |
| **Bodily Pain** | CBCR vs Control | 16.30 | 9.66, 22.93 | < 0.001 | – |
| **Bodily Pain** | HBTCR vs CBCR | 5.11 | -1.00, 11.22 | 0.101 | 0.226 |
| **General Health** | HBTCR vs Control | 22.31 | 16.07, 28.55 | < 0.001 | – |
| **General Health** | CBCR vs Control | 17.69 | 11.08, 24.29 | < 0.001 | – |
| **General Health** | HBTCR vs CBCR | 4.62 | -1.79, 11.03 | 0.158 | 0.253 |
| **Vitality** | HBTCR vs Control | 17.25 | 12.61, 21.88 | < 0.001 | – |
| **Vitality** | CBCR vs Control | 17.46 | 11.84, 23.08 | < 0.001 | – |
| **Vitality** | HBTCR vs CBCR | -0.22 | -5.31, 4.87 | 0.933 | 0.933 |
| **Social Functioning** | HBTCR vs Control | 13.14 | 8.40, 17.88 | < 0.001 | – |
| **Social Functioning** | CBCR vs Control | 18.96 | 12.76, 25.16 | < 0.001 | – |
| **Social Functioning** | HBTCR vs CBCR | -5.82 | -11.49, -0.15 | 0.044 | 0.226 |
| **Role-Emotional** | HBTCR vs Control | 15.60 | 10.96, 20.24 | < 0.001 | – |
| **Role-Emotional** | CBCR vs Control | 17.41 | 12.23, 22.59 | < 0.001 | – |
| **Role-Emotional** | HBTCR vs CBCR | -1.81 | -7.29, 3.66 | 0.516 | 0.689 |
| **Mental Health** | HBTCR vs Control | 18.46 | 14.30, 22.62 | < 0.001 | – |
| **Mental Health** | CBCR vs Control | 18.93 | 13.88, 23.97 | < 0.001 | – |
| **Mental Health** | HBTCR vs CBCR | -0.47 | -5.13, 4.19 | 0.843 | 0.933 |

*SF-36: 36-Item Short-Form Health Survey; FDR: False Discovery Rate (Benjamini–Hochberg). Scores range from 0 to 100, with higher scores indicating better health status. Adjusted differences from weighted ANCOVA with baseline as covariate. PCS and MCS are component summary scores; individual domains are subscale scores. The FDR-adjusted P value accounts for multiple comparisons across the 8 individual domains (PCS and MCS are tested as part of the hierarchical strategy and are not included in the FDR correction).*

## Supplementary Table S5. Safety Outcomes, Technical Failures, and Adverse Event Details

| **Event** | **HBTCR (n=35)** | **CBCR (n=35)** | **Usual care (n=35)** |  |  |
| --- | --- | --- | --- | --- | --- |
| 12-week MACE | 0 | 0 | 2 (5.7) |  |  |
| Emergency-department visit | 3 (8.6) | 1 (2.9) | 2 (5.7) |  |  |
| Rehospitalization | 2 (5.7) | 1 (2.9) | 3 (8.6) |  |  |
| Any exercise-related adverse event | 6 (17.1) | 4 (11.4) | – |  |  |
| Exercise-related chest pain | 2 (5.7) | 2 (5.7) | – |  |  |
| Exercise-related arrhythmia | 1 (2.9) | 1 (2.9) | – |  |  |
| Symptomatic hypotension | 1 (2.9) | 0 | – |  |  |
| Fall during exercise | 1 (2.9) | 0 | – |  |  |
| Musculoskeletal injury | 2 (5.7) | 1 (2.9) | – |  |  |
| Implementation metric (resolved-exposure cohort) | HBTCR (n=56) | CBCR (n=54) |  |  |  |
| Valid training hours | 1,473.2 | 1,271.4 | – |  |  |
| Prescribed sessions | 2,016 | 1,944 | – |  |  |
| Recorded technical-failure episodes | 34 | 0 | – |  |  |
| Technical-failure rate, % of prescribed sessions | 1.7 | 0 | – |  |  |
| Scheduled tele-consultations, mean ± SD | 10.5 ± 1.2 | – | – |  |  |

AE: Adverse Event; HBTCR: Home-Based Telerehabilitation; CBCR: Center-Based Cardiac Rehabilitation; MACE: Major Adverse Cardiovascular Events. Participant-level safety outcomes use the 105 complete cases. Implementation metrics use all patients with resolved active-rehabilitation exposure (HBTCR n=56; CBCR n=54). Usual-care patients were not enrolled in prescribed rehabilitation exercise; exercise-related event rows are therefore not directly applicable to that group. No formal comparative safety testing was performed because of sparse events.

## Supplementary Table S6. Full Multivariable Model for the Exploratory Adherence–VO₂peak Association

| **Variable** | **β Coefficient** | **95% CI** | **P Value** |  |
| --- | --- | --- | --- | --- |
| Adherence (per 10 percentage points) | 0.62 | −0.03, 1.27 | 0.062 |  |
| HBTCR vs. CBCR at mean adherence | −0.35 | −1.10, 0.39 | 0.354 |  |
| Adherence × HBTCR interaction | 0.36 | −0.42, 1.14 | 0.368 |  |
| Baseline VO₂peak (per 1 mL/kg/min) | 0.02 | −0.19, 0.24 | 0.834 |  |
| Age (per 10 years) | 0.03 | −0.61, 0.67 | 0.933 |  |
| Male vs. female | 0.06 | −1.17, 1.29 | 0.922 |  |
| BMI (per 5 kg/m²) | 0.08 | −0.52, 0.68 | 0.792 |  |
| Diabetes (yes vs. no) | −0.22 | −0.83, 0.39 | 0.471 |  |
| Education (per year) | 0.01 | −0.15, 0.17 | 0.899 |  |
| Family support (per 10 points) | −0.16 | −0.40, 0.08 | 0.193 |  |
| Digital literacy (per 10 points) | −0.12 | −0.41, 0.16 | 0.394 |  |

N=70 rehabilitation participants with complete 12-week outcomes. The dependent variable was 12-week VO₂peak change (mL/kg/min). The model used combined outcome weights and HC3 robust standard errors; adherence was mean-centered at 87.35% and scaled per 10 percentage points. Model R²=0.399; adjusted R²=0.285. This analysis is exploratory and does not establish causality; P values were not adjusted for multiplicity.
